# Supplementary material for: Are carbon emissions trading and green financial instruments synergistic? -Comprehensive quantitative research based on content analysis
Source: PLoS One. 2024 Mar 7;19(3):e0298601. doi: 10.1371/journal.pone.0298601 (PMC10919601; doi:10.1371/journal.pone.0298601)
Supplement: S1 Data — (ZIP) [file pone.0298601.s001.zip › Data/地级市Z_F工作报告.zip!/║╙▒▒/╨╧╠¿╩╨/└┤╫╘╣1⁄2╓┌║┼í╛┬φ┐╦╩2╛▌═°í┐_2005.docx]

各位代表：

现在，我代表邢台市人民政府向大会作工作报告，请予审议。请市政协各位委员和列席会议的同志提出意见。

2004年工作回顾

过去的一年，全市人民在省委、省政府和市委的领导下，高举邓小平理论和“三个代表”重要思想伟大旗帜，树立和落实科学发展观，高扬“树正气、讲团结、求发展”主旋律，营造“干事、创业、为民”浓厚氛围，较好地完成了市十二届人大二次会议确定的各项目标任务。

初步预计，全市生产总值完成628.4亿元，同比增长13.5％；全部财政收入完成46.4亿元，增长30.8％（按可比口径可达50.2亿元，增长41.4％），其中地方一般预算收入18亿元，增长21.1％；全社会固定资产投资完成278亿元，增长27.7％；社会消费品零售总额完成180亿元，增长15.5％；实际利用外资完成1.07亿美元，增长85.5％；城镇居民人均可支配收入6695元，增长9.3％；农民人均纯收入3003元，增长10.1％；城镇新增就业岗位3.65万个，城镇登记失业率3.9％；人口自然增长率6.03‰。科技、教育、文化、卫生、体育等社会事业全面进步。

一年来，我们主要做了以下几方面工作：

（一）积极贯彻中央宏观调控政策，国民经济实现快速协调健康发展

坚持积极的、有作为的工作方针，把贯彻中央宏观调控政策与邢台实际结合起来，全面理解，准确把握，坚定信心，顺势应变，坚持调控当中讲机遇,发展当中讲速度,统筹当中讲投入，求快当中讲求实，努力做到符合产业政策和调控方向的投入不减少，有质量、有效益的速度不减慢，技术含量高、市场前景好的项目不放松，保持了全市经济快速平稳发展。

认真落实各项惠农政策，促进了农业增效、农民增收。认真贯彻中央1号文件精神，组织干部深入农村开展宣讲活动，坚决落实以“两减免、三补贴”为重点的各项惠农政策，全市农业税率降低3.01个百分点，减轻农民负担1.52亿元；6266万元粮食直补、农机具补贴和良种补贴资金全部兑付到户，调动了广大农民生产积极性。全年粮食总产324.4万吨，比上年减少4.9％；棉花总产18.1万吨，增长48.4％；肉、蛋、奶产量分别增长3.6％、2.7％、18.1％。农业结构日趋优化，优质专用小麦、优质玉米、无公害蔬菜、设施蔬菜种植面积增加。畜牧业占大农业的比重提高1.9个百分点。大力扶持龙头企业和专业合作组织，农业产业化经营率提高1.2个百分点，新增年销售收入5000万元以上龙头组织4家、省级重点龙头企业5家，中旺集团跨入国家级重点龙头企业行列。加强全市动物防疫体系建设，成功阻击高致病性禽流感疫情发生。

加强经济运行监控和调节，质量和效益明显提高。全年规模以上工业增加值完成171亿元，增长23.8％；实现利税58.5亿元，增长31％。全市全部财政收入占GDP比重、税收占财政收入比重实现了“双提高”。财政收入超亿元的县（市）达6个。其中，沙河市、邢台县财政收入实现翻番。

积极优化投资结构，重点项目建设取得明显进展。坚持有保有压，有所为有所不为，26个不符合政策规定的项目分别被停建或取消立项；为16个限期整改项目补办前期审批、土地征用、环境保护等各项手续；积极支持符合国家产业政策、有市场有效益的重点项目建设，做到了宏观调控之年项目投资规模不缩小，项目数量不减少。全年重点项目建设完成投资137.6亿元，增长42％，是历史上投入最多的一年；新上投资亿元以上的项目81个，其中投资10亿元以上的项目10个。内邱县建滔焦化、邢台县旭阳焦化一期等62个市级以上重点项目竣工投产。邢钢线材加工基地去年底开工建设，核心企业投资4亿多元的新光线材制品项目建设迅速，可望今年4月份投产。投资23亿元的国泰发电2×30万千瓦项目，提前一年开工建设，第一台机组预计今年6月份投产。邢临、青银高速分别完成投资8.2亿元和9.3亿元，建设农村公路2300公里，新增通油路村1643个，全年共完成公路投资28亿元，增长50％。完成了滏阳河中游洼地治理二期续建工程，治理山区水土流失面积130平方公里。

（二）坚持求深入求实效，重点工作稳步推进

按照“往深里想、往实处做”的要求，努力在深入中实现突破，在求实中加快发展。

各项改革继续深入。坚持以产权制度改革为核心，以职工身份转换为关键，以健全法人治理结构为中心，加快国企改革步伐。全市国有企业累计完成改制935户，改制面达96.2％。粮食流通体制改革扎实推进，基本完成了省下达的粮食购销企业布局调整和职工身份转换任务。加强国有资产监督管理，规范了改革操作程序和产权交易行为。同时，财政体制、投资体制、公用事业等项改革也都取得不同程度的进展。

民营经济发展壮大。坚持“六放”方针，完善扶持政策，在市场准入、土地供应等方面，进一步落实了国民待遇；引导民营企业参与国企改革、扶贫开发和产业化经营，实现互利双赢；大力推进产业和企业的整合提升，新增通过省认定的县域特色产业3个，总数达到18个；20家企业进入全省百强，华龙、奎山、德龙进入前10名。全年民营经济增加值增长16.7％，上缴税金增长47.8％。

对外开放步伐加快。先后举办了深圳和香港项目推介会、与江浙沪和京津经济文化合作论坛。不断改进招商方式，提高招商效果。全年新注册三资企业25家，注册资本2.1亿美元。外贸出口结构进一步优化，关键设备和技术引进力度加大，完成进出口总值5.6亿美元，增长58.6％。其中出口4.3亿美元，增长42.5％。

城镇建设力度加大。完成了城市总体规划，对14个县（市）总体规划进行修编，编制32个建制镇的规划。进一步加大了市区建设力度，组织实施了路网建设、新区开发、旧城改造、中心区美化包装等项工程，新建、拓宽、延伸道路13条，对“五路、十六街”进行了亮化改造，对5条街道进行了硬化，对达活泉公园进行了大规模的改造，完成了市污水处理厂二期工程，投资1.4亿元的市垃圾处理厂开工建设，新增绿地328万平方米，绿化覆盖率达到34.5％。各县（市）新建和扩建了一批道路、排水、垃圾处理等基础设施项目。全市城镇化水平提高1.6个百分点。

（三）社会事业全面进步，可持续发展能力进一步增强

教育改革继续深化，各级各类教育协调发展。桥东区、邢台县等9个县（区）顺利通过省第二、三轮“普九”复查验收，加快推进农村中小学布局调整。81所农村中小学危房得到改造，新增校舍9万平方米。新增省级示范性高中4所。职业教育发展迅速，培训转移农民2.1万人。科技创新与推广步伐加快，完成科技成果35项，推广科技成果38项。重视文化事业和文化产业发展，加快了基层文化设施建设，创作了一批反映时代和生活的文艺作品，广播电视覆盖率进一步提高，群众性文化活动开展广泛，丰富了城乡文化生活。大力开展计划生育集中整治活动，完成了人口控制目标。加强公共卫生体系建设，市传染病医院和3个县级传染病医院及16个县级疾控建设项目已经完成，乡镇卫生院标准化建设开始启动。全民健身活动广泛开展，竞技体育取得较好成绩。狠抓城市环境综合治理，市区空气质量进一步好转，新增二级及好于二级天数47天；加大饮用水源保护和工业废水治理，饮用水源水质100％达标。大力发展循环经济，生态环境进一步改善。根据国务院、省政府统一部署，启动了第一次全国经济普查。严格执行事业单位机构编制下管一级的规定，全市各类事业编制保持了零增长。民族宗教、外事侨务、妇女儿童、地震、气象、档案、地方志等工作，也都取得了新成绩。

（四）高度重视民生问题，认真实施十项“民心工程”

坚持以人为本，从解决人民群众最现实、最关心、最直接的问题入手，认真实施了十项“民心工程”。实行积极的就业再就业政策，完成了年度目标任务。9914名下岗失业人员实现再就业，安排“4050”人员2000名。做好“两个”确保和城镇“低保”工作，推进“三条保障线”衔接。国企下岗职工基本生活保障金和企事业单位离退休人员养老金全部按时足额发放。企业退休人员月增生活补贴20元、养老金39.5元。养老、失业、医疗、工伤保险扩面进展顺利。城镇3万名低保对象实现了应保尽保。完成了5个村、107户移民搬迁，基本解决350个贫困村的群众温饱问题。对农村特困户和五保户全面实行了定期定量救济。市区新增集中供热能力200万平方米，提高330％；新增燃气用户1万户，总户数提高20％。全市农村新建沼气用户4.4万户，解决7万人和近1万头大牲畜饮水困难。

（五）大力推进政府职能转变，不断优化发展环境

切实加强政府自身建设，强化社会管理和公共服务，把政府管理经济职能转到主要为市场主体服务和创造良好发展环境上来。

自觉接受人大、政协和群众的监督，认真办理人大代表议案、建议和政协委员提案。去年，共办理人大代表建议329件、政协委员提案505件，有10名政府组成部门主要负责人接受了市人大常委会述职评议。强化了政府内部监察、审计等专项监督。一些重大事项决策，主动听取专家、学者和民主党派、工商联、无党派人士的意见。认真贯彻《行政许可法》，大力推进行政审批制度改革，清理规范性文件1300余件，市本级取消21项行政许可。加强廉政建设，严厉查处了一批违法违纪案件。

全面落实《关于进一步优化经济发展环境的若干规定》，政府机关实行首问负责制、领导带班制，推行“一厅式”办公、“一条龙”服务。开展民主评议活动，加强行政监察，提高行政效能。积极为企业和投资者排忧解难。加快诚信邢台建设，整顿规范市场经济秩序，发展环境明显改善。

加强爱国主义教育基地建设，深化未成年人和学生思想道德教育，认真做好“双拥”工作，广泛开展群众性精神文明创建活动。大力推进文明生态村建设，首批60个市级和220个县级文明生态村达到创建要求。积极推进国防动员实案化建设，民兵预备役工作取得新进展。实行安全生产责任制，严肃查处各类安全事故。高度重视信访工作，强化社会治安综合治理，严厉打击各类违法犯罪活动，维护了社会稳定。

各位代表，过去一年所取得的成绩是省委、省政府和中共邢台市委正确领导的结果，是全市人民同心同德共同努力的结果！在此，我代表市人民政府，向广大工人、农民、知识分子、各级干部和社会各阶层的建设者，向人大代表、政协委员，向各民主党派、工商联、无党派爱国人士和人民团体，向驻邢人民解放军、武警官兵，向中省直驻邢各单位，向所有关心和支持我市发展的海内外朋友，致以崇高的敬意和衷心的感谢！

各位代表，面对当前好的形势，我们也清醒地看到，经济和社会生活中还存在不少矛盾和问题。产业结构不优、增长方式粗放、工业化水平低、体制改革滞后、经济总量小<城乡及县市发展不平衡等深层次矛盾还没有解决。粮食增产和农民增收基础不稳固，去年农民收入大幅增长，主要得益于以粮食为主的农产品价格回升和各项惠农政策的落实。随着农产品价格涨幅回落，农民增收空间将逐步缩小。部分市本级企业实力弱、改制慢、效益低。就业再就业形势严峻，资源制约问题突出，环境压力加大，煤电运油紧张状况依然没有缓解。社会治安面临新情况，重大安全生产事故时有发生，发展环境不尽如人意，特别是一些政府部门和工作人员中官僚主义、形式主义和腐败问题依然存在。对此，我们将高度重视，认真加以解决。

邢台必须、也有条件和能力发展的更快一些、更好一些。政府工作任重而道远。我们应倍加珍惜难得的发展机遇，倍加珍惜良好的发展局面，倍加珍惜现在的发展氛围，居安思危，切实增加责任意识和忧患意识，为加快邢台发展竭尽全力，决不辜负人民的期望。

2005年工作总体要求及主要任务

今年，是全面实现“十五”计划目标、衔接“十一五”发展的重要一年。做好今年的工作至关重要。纵观全局，机遇与挑战并存，希望与压力同在。我们自己与自己比发展速度比较快，但横向比，在全省的后进位次没有改变，“不进则退，慢进亦退”的形势没有改变。我们在发展，兄弟市也在发展，而且有些经济总量比我们大、经济结构比我们优的市，发展速度和投入规模都超过我们。我们只有发展速度更快，拼搏精神更强，发展环境更优，才有可能赶超先进，把邢台推入大发展的快车道。抢抓机遇，实现更快更好发展，是当前和今后一个时期全市工作的主题，也是全市670万人民的根本利益所在和迫切要求。

今年政府工作的总体要求是：以邓小平理论和“三个代表”重要思想为指导，认真贯彻党的十六大和十六届三中、四中全会、省委六届六次全会和市委六届七次全会精神，以抢抓机遇、实现更快更好发展为主题，以科学发展观为统领，以结构调整为主线，以项目建设为龙头，带动经济增长方式转变，带动重点工作突破，带动各项社会事业发展，带动扩大开放，带动环境优化，着力构建和谐邢台，努力开创跨越式发展和全面建设小康社会新局面。

我市今年经济社会发展的主要预期目标是：生产总值增长12％以上；全部财政收入增长13％以上；城镇固定资产投资增长30％以上；实际利用外资增长16％以上；城镇居民人均可支配收入增长8％以上；农民人均纯收入增长6％以上；居民消费价格总水平上涨4％；城镇登记失业率控制在4.5％以内；人口自然增长率控制在6.4‰以内。

完成全年目标任务，必须坚持以科学发展观统领全局，一切从邢台实际出发，因地制宜、因时制宜地把科学发展观贯穿于各个方面；坚持抢抓机遇、实现更快更好发展这个主题，靠发展解决前进中的困难和问题，在提高质量和效益的前提下，发展速度越快越好；坚持以结构调整为主线，加大投入力度，切实抓好一批能够影响和带动全局的重点项目，促进经济增长方式转变；坚持以人为本，落实五个统筹要求，优化发展环境，高度关注民生，切实维护好、实现好、发展好人民群众的根本利益；坚持进一步解放思想，创造性地开展工作，以改革为动力，破除体制性障碍，力争在一些重要领域和关键环节实现新突破；坚持求真务实，提高抓落实的能力、解决矛盾的能力、破解难题的能力和干实事干成事的能力。今年着力抓好以下十个方面的工作：

（一）大力开展“项目年”活动，拉动经济持续快速发展。坚持以项目为支撑，围绕调整产业结构，转变经济增长方式，加大项目建设力度，努力做到项目建设规模比往年大，数量比往年多，质量比往年好，推动经济更快更好地发展。

抓好已确定的重点建设项目。全年在建和新开工重点项目157个，总投资500.5亿元，年度投资151.6亿元，增长24.5％。其中，超亿元项目98个，超10亿元12个。对已开工在建的项目，如邢钢线材制品加工基地、沙河玻璃城、德龙二期、内邱建滔焦化二期、旭阳煤化工、隆尧东方食品城、宁晋单晶硅园区、国泰发电6期、市垃圾处理厂等项目，加强协调调度，及时解决存在问题，确保建设进度。对南宫“千喜鹤”肉制品加工、新河天津造纸厂、红星技改二期、国泰发电7期、医专附属医院等重大项目，重点推进，尽快开工建设。

进一步搞好项目谋划和储备。发挥比较优势，紧紧围绕国家产业政策支持重点、改造提升传统产业、做强优势骨干企业，谋划一批立县、立市重大项目，并力争多列入国家和省的重点项目笼子，做到项目建设不停步、不断档。尤其对已初露端倪的项目，要紧盯、紧跑，锲而不舍，抓住不放。

创造性地解决项目建设的瓶颈制约问题。在项目建设用地上，既坚决执行国家的土地政策、不撞“红线”，又要在解决用地问题上有所作为。按照国家政策要求，继续采取用好已批土地，清理批而未用和低效利用土地，盘活破产和停产企业存量土地，利用荒坡、荒地、荒滩土地，积极争取国家和省支持等措施，千方百计增加重点项目建设用地的有效供给。同时，提高项目容积率，提高土地集约利用水平。在解决项目建设资金问题上，要深化投融资体制改革，既发挥银行的主渠道作用，又积极支持有实力的优质企业发行股票和债券，鼓励企业进行产权和股权转让等方式吸引国内外资金，通过招商引资、激活民资，努力实现项目投资主体多元化。

努力营造推进项目建设的良好环境。坚持从实际出发，讲规律、求实效，敢闯敢干敢为人先，不能安贫乐道，不思进取，不能前怕狼后怕虎，患得患失，要有一种事业不发展、人民不富裕，寝食不安、无地自容的责任感，以良好精神状态推进项目建设。进一步完善奖惩机制，把项目建设的成效作为检验政府工作的重要依据，在项目建设中考验干部，在项目建设中锻炼培养干部。要围绕大局形成合力。政府各部门要紧紧围绕推进项目建设、加快发展这个大局去开展工作，参与项目建设，支持项目建设，服务项目建设，不当旁观者，都做“运动员”；对项目建设中遇到的困难和问题，不要轻易说这也不行、那也不行，而是要积极帮助出主意、想办法、当参谋，绕开障碍，破解难题，尽一切努力促成，为项目建设创造好的氛围。

（二）切实做好“三农”工作，促进农业增效农民增收。继续把解决“三农”问题作为全局工作的重中之重，认真落实各项扶持政策，促进农业持续增效，农民继续增收，农村经济全面发展。

打特色牌，进一步优化农业结构。按照“粮食调优、畜牧调强、林果调特、蔬菜调大”的思路，继续抓好畜牧、蔬菜、果品三个主导产业发展，努力做大做强。同时，大力培育食用菌、林木、饲料饲草三个新兴产业，积极发展设施农业、高效农业、出口农业、生态农业，全面推进农林复合经营，提高农业比较效益。

培育龙头，加快产业化经营步伐。按照“抓龙头、带基地、兴产业、促增收”的原则，谋划引进和建设一批具有核心带动作用的产业化“龙头”项目，鼓励有实力的企业投资创龙头，通过对外开放引龙头，培植市场育龙头，进一步完善龙头企业与农户的利益联结机制。全年发展投资额1000万元以上农业产业化重点项目23个，总投资8亿元。重点抓好旭瑞三期、三鹿二期、益发板栗深加工、国宾食用菌示范园等项目。到年底，新增年销售收入5000万元以上的龙头企业5家，农业产业化经营率比上年提高1个百分点。

加强基础建设，完善服务体系。围绕完善基础设施和提高农业综合生产能力，投资11.6亿元，搞好水库除险加固、滏阳河中游洼地治理、节水灌溉、农业综合开发等工程；建立和完善良种繁育、质量检测等“六大体系”，大力实施“农民培训”和“科技示范”两大工程，提高农民科技意识和市场意识，增强农产品市场竞争能力；抓好太行山区绿化和生态建设，搞好防疫、防汛、防火、防雹、防虫工作，提高农业抵御自然灾害能力。

发展劳务产业，实施农民转移工程。认真落实培训补助、费用减免、权益保障、就业激励和社会保险五项优惠政策；组织好大型劳务输出和劳动力专场、专岗洽谈会，努力为农民进城务工、进入非农产业领域创造良好条件。同时，多措并举、“分船渡海”，加快农村劳动力向特色产业转移、向民营企业转移、向城镇转移。到年底，全市有组织的转移农村剩余劳动力12万人。

落实惠农政策，减轻农民负担。认真落实中央和省各项惠农政策，进一步深化农村税费改革，落实“两减免、三补贴”，农业税率再降两个百分点，三个国家级贫困县全部免征农业税；继续实行对种粮农民的直接补贴，增加良种补贴和农机具补贴，进一步调动农民生产积极性；扎实推进粮食流通体制改革，维护正常粮食流通秩序；开展物价检查，规范涉农收费和农资市场专项治理，进一步减轻农民负担。

（三）调整优化产业结构，培育壮大主导产业和骨干企业

改造提升传统产业。积极运用先进适用技术改造提升我市有比较优势的羊绒、玻璃、自行车零件、轴承、电缆、板材等传统产业，改进工艺装备，创新生产技术，推动产业升级，提高企业竞争力；充分发挥骨干企业的辐射带动作用，通过联合、重组、兼并等形式，用增量资产优化存量资产，在每个行业扶持一批骨干企业率先发展，做大做强，发挥领头羊的作用，带动整个产业升级；鼓励企业向工业园区集中，形成产业集群，加快推进清河国际羊绒科技园区、沙河金百家工业园区、临西轴承大世界、隆尧东方食品城、宁晋单晶硅园区等工业园区的建设，构建平台，筑巢引凤，引进一批国内外大企业、大公司，打造知名园区，增强产业聚集效应。

培育壮大骨干企业和支柱产业。制定实施钢铁、建材、机械制造、煤化工、电力、纺织服装、医药、食品加工等八大主导产业的专项发展规划，分类指导，力争用2－3年时间，使这些产业成为销售收入超百亿元以上的主导产业。以优势产业为依托，以骨干企业为主体，聚合各种生产要素，加大扶持力度，全市抓好90个重点项目，上品种、上质量、上效益，力争用3－5年时间使邢钢、邢矿、邢机、晶牛、德龙、华龙、中旺、国泰、旭阳、建滔、吉泰特钢等企业，成为年销售收入超50亿元或100亿元以上的大型骨干企业集团，带动整个产业和一批中小企业发展。大力实施名牌战略，积极培育壮大支柱产业和骨干企业，加快发展高新技术产业，以名牌产品开拓市场，提高企业竞争力，以名牌产品做大产业、做强企业。

强力推进国企改革。抓重点、攻难点，不回避、不退缩，力争在产权制度改革上有大的突破，在完善法人治理结构上有新的成效，在国有经济布局调整上有明显进展，基本实现“两增、两减、两分”，基本完成市属39家国有工业企业改制，县市区国有企业改制全部结束。把企业改制和开放招商引进新的生产要素、救活和做大做强一批企业结合起来，加快国有产权流动重组，支持具备条件的企业实现整体上市。建立规范的现代企业制度，健全公司治理结构。完善国资监管体系，防止国有资产流失，维护出资人和职工合法权益。

加快发展循环经济。按照减量化、再利用、资源化的原则，广泛开展建设节约型经济和节约型社会活动。加快推广节能降耗技术，发展节水农业，抓好钢铁、水泥、化工、造纸、建筑等行业节能、节水技术改造，提高资源利用效率。对旭阳、建滔、邢钢大量煤气资源白白烧掉的问题，环保、城建等部门要协调有关用热大户使用煤气，以节约能源，清洁城区环境。加强矿产资源开发规划和管理，坚决制止滥采乱挖和浪费现象。推行清洁生产，发展环保产业，努力降耗减污，提高环保水平。

（四）进一步发展民营经济，增强经济发展活力。充分发挥民营经济在促进增长、扩大就业、活跃市场、增加财政收入等方面的重要作用，毫不动摇地鼓励、支持和引导民营经济加快发展。全年民营经济增加值增长16％以上，上缴税金增长18％以上，固定资产投入增长16％以上。

认真贯彻国家鼓励、支持和引导非公有制经济发展的政策，为非公有制经济创造平等竞争、一视同仁的法制环境、政策环境和市场环境。进一步拓宽市场准入行业和领域，凡法律法规未禁入的，非公有资本均可进入；凡外资可进入的，国内非公有资本均可进入。着力解决中小民营企业资金短缺、人才匮乏、创新乏力等问题。推进银企合作，加快中小企业信用担保体系建设，扩大规模，规范运作，防范风险。积极探索企业债券、上市融资、股权融资等直接融资方式，帮助有条件的企业上市。继续扶持一批规模型、成长型民营企业，培育一批名牌产品，支持一批中小企业产业集群加快发展。鼓励民营企业通过多种有效形式参与国企改革、扶贫开发和基础设施建设，积极发展民营科技型、外向型、劳动密集型企业。切实加强民营企业家队伍建设，对做出突出贡献的民营企业家，在政治上给荣誉，在社会上给地位，形成尊重人才，崇尚创业的良好风尚。加大治理“三乱”力度，减轻企业负担。

引导民营企业适应宏观调控新形势，在企业制度、经营模式、增长方式等方面加快实现转变。加强政策信息和行业规划引导，帮助民营企业把握投资方向，认真执行国家产业政策和技术、环保、质量、安全等市场准入标准。民营企业要依法经营、照章纳税、诚实守信、规范劳动关系，保障职工合法权益。

（五）统筹城乡发展，努力提高城镇化水平

完善城镇规划。按照优先发展市区，积极发展次中心城市和县城，有选择地发展重点镇和建制镇的思路，完成市域城镇体系规划和城市总体规划修编，进一步明确功能定位，加强分类指导。搞好城镇总体规划和土地利用规划修编的衔接，使之相互协调。中心城市，围绕建设冀南重要中心城市、新型工业城市和生态旅游城市的功能定位，实施外延扩区，内涵挖潜，完善分区，加快发展，大力抓好壮大产业、创建省级园林城市、申报和建设历史文化名城工作，强化聚集辐射能力，发挥领跑作用。次中心城市，发挥沙河、清河、南宫、宁晋区位和产业优势，加强与中心城市的规划衔接，逐步完善功能，发展成为环境良好居住舒适，有较强吸引力的中小城市。其他县城和小城镇，积极发展特色经济，增强产业和人口的集聚能力。

加强基础设施建设。市区按照“新区开发和旧城改造并重”的思路，组织实施“一二四六八”工程，即“一环”（环路建设），“两河”（七里河和围寨河综合开发治理），“四街”（北迎宾线、东迎宾线、南出市口和新区景观轴四条街道建设），“六园”（森林公园、郭守敬文化公园、仲毅公园、紫金公园、古城墙遗址公园和达活泉公园扩建六项园林建设），“八大工程”（路网建设、供热供气、垃圾处理厂和污水处理厂续建、市场建设、住宅开发和生活小区治理、中心城区改造、“城中村”改造、城网改造和城通信息管网工程等八项城市基础设施建设）。同时，搞好县城和重点镇建设，发挥区域中心的带动作用。城镇化水平比上年提高2个百分点。

深化城市经营管理。整合城镇各类资源，运用股权转让、招标拍卖等方式，盘活存量资产，大力吸引民间资本、社会资本和外资投入城镇建设，尽快走上投资多元化、建设多渠道的轨道。推进城市管理综合执法、环卫、公交等体制改革。

（六）强力推进对外开放，借助外力促发展。坚持以开放促改革、促发展，不断扩大开放领域，充分利用市内市外、国内国外两个市场、两种资源，加大招商引资力度，千方百计利用外资上一批大项目、好项目。

突出抓好招商引资。坚持跳出区域看优势，打破界限看市场，突破区划育产业，发挥优势，主动出击，开展全方位、多层次、宽领域的招商引资活动。抓住重点招商领域，鼓励外商投资改造传统产业、发展高新技术产业、现代农业、现代服务业、环保产业和基础设施产业；不断创新招商方式，大力开展多种形式的招商活动，注重招商实效，提高项目履约率和外资到位率；重视寻找战略合作伙伴，加强与国际国内大公司、大集团的合资合作，重点引进基地型、龙头型、科技型大项目。当前，着力抓好一批投资1000万美元以上外资项目和投资亿元以上内资项目。

加强区域经济合作。抓住南资北上机遇，巩固和扩大与沿海发达地区经贸合作；利用国家启动京津冀都市圈规划的有利时机，积极参与京津产业分工协作，在与京津的产业对接中谋求发展；进一步深化与中原经济圈城市间的合作和交流，实现优势互补；落实全省“一线两厢”战略，培育壮大具有邢台特色的产业集群，形成南厢新的增长极；充分利用京、津、沪等地高等院校和科研院所的教育、科技、人才优势，加大“产学研”对接力度，引进智力和急需高级专业人才，全面增强科技创新能力。

搞好外经外贸。切实做好入世后过渡期应对工作，积极发展对外贸易，由过去注重出口创汇转移到注重拉动经济增长上来，尤其是抓住今年取消纺织品配额的机遇，发挥我市纺织服装、床上用品等行业优势，扩大产品出口，开拓国际市场。同时，增加短缺原材料、关键技术和重大装备进口，促进企业技术改造，发展高新技术产业。

（七）加快发展服务业，培育新的经济增长点

加强市场建设。制定城乡市场和商业网点建设规划，搞好布局结构调整，加快城乡市场体系Ｉ琛；嘤徒ㄉ枳ㄒ蹬⑹谐。氐惴龀帜舐酱迳堂吵恰⑼毓喜伺⒌却笮妥ㄒ凳谐。咸嵘傥髦岢写笫澜纭⑵较绾庸琶碜孕谐导傲闩浼⑶搴友蛉拗破返忍厣谐　；⒄棺时尽⑼恋亍⒓际酢⑷瞬拧⒗投Φ壬厥谐。∪骋弧⒖拧⒕赫⒂行虻南执谐√逑怠Ｈ旯烫岣咭谠陨鲜谐?1个，新改扩建市场17个；商品交易额达134亿元，增长15％以上。

大力推进流通现代化。加快发展连锁经营、集中配送和电子商务。做大做强新亚、家乐园、华天等一批连锁龙头企业，使网点向社区、乡镇、农村延伸。新建邢台综合物流园区配送中心，抓好石油、烟草、糖酒等三大配送网络建设，提升现代流通方式的经营比重。研究发展我市医药批发市场，加快商业、粮食、物资企业改革，搞活传统流通业。

积极发展以旅游为重点的现代服务业。搞好全市旅游总体规划，加大投入，整合资源，完善基础设施建设，加大市场推销力度，着力打造太行奇峡群、崆山白云洞、鹊山风景区等一批旅游精品，构筑以市区、邢台县、内邱、临城为中心的一日游或多日游黄金旅游线路，促进旅游业快速健康发展。大力推进信息化建设，建立资源共享的信息高速公路和信息数据库。积极发展会计、律师、评估、咨询、公证、房产交易等中介服务组织。鼓励创办家政、托幼、养老、保健等社区服务业，推广和规范物业管理，促进社区服务网络化和产业化。

（八）切实加强财税金融工作，提高支持经济发展能力。以建立和完善公共财政体制为目标,全面推进依法治税和公共财政体制建设，为培植财源、促进发展、维护稳定发挥积极作用。

培植壮大财源。通过财政贴息、补贴、担保等方式，充分发挥财政资金支持经济的“乘数效应”，培植壮大财源，支持发展。通过完善财政体制、转移支付和支持县乡财政体制改革等，大力发展县域经济，逐步解决县乡财政困难问题。

强化税收征管。依法加大税收征管力度，规范非税收入的执收行为，一方面做到应收尽收，严防跑冒滴漏和压延税款，另一方面要严禁虚收形成新的水份。同时，搞好预算管理、国库管理、政府采购和“收支两条线”四项改革，推进财政体制和管理机制创新。

节约财政支出。按照“一要吃饭，二要建设”的原则，优化支出结构，合理安排预算，严格支出顺序，强化预算约束。牢固树立过“紧日子”的思想，勤俭办一切事业，压缩一切不合理开支，狠刹奢侈浪费歪风。建立支出效益评价、考核监督和责任追究制度，严肃财经纪律，杜绝财政资金脱离监管、挤占挪用等问题的发生。加强机构编制管理，严格控制机构编制增长，切实维护机构编制的严肃性。

与此同时，大力加强金融工作，进一步优化信贷结构，引导贷款投向，优化金融服务，加大对经济发展的支持力度。按照省政府统一部署，切实抓好农村信用社改革，重点搞好增资扩股，大力清收盘活不良贷款，按期达到国家规定标准，确保国家扶持资金落实到位。

（九）协调发展社会事业，增强可持续发展能力

加快科教兴市步伐。加快科技进步与应用推广，实施农业科技创新、用高新技术改造传统产业、科技引联、特色产业升级四大重点科技项目，完善区域科技创新体系，增强科技对经济发展的促进作用。全年推广重大科技成果40项，主要农作物良种覆盖率达到98％。优先发展教育事业，认真落实以县为主的管理体制，加强素质教育和基础教育，做大做强职业教育，加快发展高等教育，走“建名校、强职教、扩高教”之路。同时，抓好人才开发、引进和使用工作，为经济社会发展提供人才保障和智力支持。

扎实推进文化建设。增加文化事业投入，发展文化产业，打造郭守敬、扁鹊等文化品牌，搞好文物保护，抓好历史文化名城申报工作，开展丰富多彩、健康向上的群众文化活动，积极推进文化大市建设。加大“扫黄打非”力度，培育健康有序的文化市场。坚持人口管理目标责任制，强化基层基础工作，搞好集中整治，稳定低生育水平。构建全民健身服务体系，广泛开展全民健身运动。认真做好第一次全国经济普查工作，编制“十一五”经济社会发展规划。进一步做好双拥工作，搞好国防教育，推进民兵预备役工作，加强国防后备力量信息化建设。积极支持工会、共青团、妇联等群众团体的工作，继续做好民族、宗教、外事、侨务、档案、地方志、防震减灾等各项工作。

促进经济社会与生态良性循环。合理开发利用国土资源，提高对经济社会发展的保障能力。围绕改善环境质量和控制污染物排放，突出抓好水和大气污染防治，全面加强工业污染防治、城市环境综合整治、生态环境保护，大力发展环保、节能、节地新型建筑体系。积极开展“环保模范城”创建活动，努力把我市建成经济繁荣、环境优美的新型城市。

加强社会主义精神文明建设。以庆祝抗战胜利暨纪念邢台解放60周年活动为契机，广泛开展爱国主义、社会主义、集体主义教育，大力培育和弘扬民族精神。加强社会公德、职业道德和家庭美德教育，突出抓好未成年人思想道德建设。坚持典型带动与整体推进并重，深入开展文明城市、文明生态村、文明社区和文明单位创建活动。扎实有效地推进“四五”普法和依法治市工作。深入开展“诚信邢台”建设，倡导健康文明生活方式。

（十）切实解决群众生产生活中的突出问题，构建和谐社会。坚持以人为本，高度关注民生，认真办好一批与群众生产生活密切相关的实事，切实解决广大人民群众生产生活中的困难。

认真做好就业和再就业工作。坚持在发展中解决就业的原则，逐步确立有利于扩大就业的经济结构和增长模式，注重发展就业容量大的服务业、中小企业和劳动密集型产业，形成更多的就业增长点。充分利用发放小额贷款、税费减免等优惠政策、扶持下岗失业人员、被征地农民和大中专毕业生的就业和再就业，鼓励自谋职业、自主创业、灵活就业。加大就业再就业投入，完善公共就业服务和再就业援助制度，积极开展就业培训，加强人力资源市场建设。全年新增就业岗位2.6万个。

着力提高社会保障能力。加快建设与经济发展水平相适应的社会保障体系。做好“三条保障线”的衔接，健全完善失业保险制度和城镇居民最低生活保障制度，确保企业离退休人员养老金按时足额发放。依法扩大养老、失业、医疗、工伤社会保险覆盖面，稳步推进城镇职工基本医疗保险制度改革。高度关注困难群众生产生活问题，发展社会救济和社会福利事业，完善对低收入者救助制度。落实帮扶措施，帮助困难家庭解决就医看病、子女上学、住房等实际困难。

扎实推进“民心工程”。从解决人民群众最现实、最关心、最直接的问题入手，继续实施“民心工程”。解困助残工程，完善农村特困户定期定量救济制度，救济面达3％以上；实施“星光计划”，60％的县建立综合福利机构；筹建残疾人综合服务中心。扶贫开发工程，力争3年内基本解决第二批182个贫困村的群众温饱问题。农村路网工程，投资3.5亿元，建设农村公路2257公里，新增通油路村880个，力争实现村村通油路、村村通客车。同时，研究建议乡村公路养护机制。危房改造工程，改造农村中小学危房3.5万平方米，新建校舍8.7万平方米。特困生救助工程，设立专项资金，资助农村困难家庭学生就学和高校学生助学贷款贴息。公共卫生工程，搞好重大疫病预防控制，全面提高城乡医疗水平和公共卫生事件应急处置能力。公共基础设施建设工程，完成5个县文化馆和图书馆建设；新增有线电视用户3万户，扩大电视网络覆盖面；建设市妇女儿童活动中心。食品安全工程，清理整顿定点屠宰，加强肉类市场监管，使群众吃上放心食品。人畜饮水工程，年内投资3000万元，解决155个村的人畜饮水困难。社会平安工程，强化社会治安综合治理，严厉打击各种刑事犯罪活动，高度重视安全生产工作，把各项安全措施不折不扣落到实处。确保政治安定，社会稳定，建设平安邢台。

切实加强和改进政府工作

牢记立党为公、执政为民的宗旨，大力推进政府自身建设特别是行政能力建设，切实做到为民、务实、高效、清廉。

提高依法行政能力，实行科学民主决策。坚持依靠科学的态度、民主的方法管理经济社会事务，积极推进政府管理理念、管理机制和管理方式创新，逐步完善公共参与、专家论证、政府决策相结合的决策机制。加大政务公开力度，对涉及经济社会发展全局和群众切实利益的重大事项，广泛征询意见，保证群众依法行使民主参与和民主监督权利。自觉接受人民代表大会及其常委会监督，接受政协民主监督，认真听取民主党派、工商联、无党派人士及各人民团体意见，接受新闻舆论和社会公众监督，加强政府内部监督。全面贯彻《行政许可法》和《依法行政纲要》，带头维护宪法和法律权威，严格按照法定权限和程序行使权力、履行职责，认真解决有法不依、执法不严、多头执法和执法扰民等群众反映强烈的问题，把政府各项行政行为纳入法制化轨道。

转变政府职能，优化发展环境。在继续履行好经济调节和市场监管职能的同时，要加快政府职能向促进市场发育、实现制度创新转变。更加注重强化社会管理和公共服务职能，提高处置自然灾害、事故灾难、公共卫生和社会安全方面突发事件的能力。充分发挥市场机制作用，凡是市场能运作的，政府不包揽；凡是企业能自主经营的，政府不干预；凡是该社会办理的，政府不插手，努力解决政府管理经济工作中的“越位”、“错位”和“缺位”问题。深入落实优化发展环境的各项政策措施，着力解决思想观念、办事效率、管理公开公正、司法保障及社会诚信等方面的突出问题。积极推行政务公开、厂务公开、村务公开等制度，增强政府工作透明度，让行政权力在阳光下运行。加快电子政务建设，提高行政效率和市场监管、公共服务能力。建立健全发展环境评价考核机制，努力营造创业宽松、社会文明、人居安全的发展环境。

改进工作作风，打造群众满意的服务型政府。服务群众，执政为民，是人民政府的根本职责。各级政府及其工作人员，要 把转变作风落实到建设服务型政府上来，落实到提高办事效率上来，落实到为企业和群众解决实际问题上来。坚持察实情，办实事，求实效，大兴求真务实之风，不图虚名，不务虚功，反对浮躁虚夸、急功近利和铺张浪费，力戒官僚主义、形式主义，坚决压缩会议和文件，减少迎来送往和检查评比活动；按照“为民、务实、高效”的要求，创新服务理念，改进服务方式，上半年建起“邢台市政务服务中心”，实行“一厅式”办公、“一条龙”服务，简化办事程序，提高办事效率，做到能马上办的事立即办，能跑一趟办成的事不让群众跑两趟。继续开展“企业评部门”活动，努力克服政出多门，程序繁琐，推诿扯皮，办事拖沓的问题；进一步完善促进各项工作落实的教育机制、责任机制、激励机制和督查机制，动真招，用实劲，敢较真，确保令行禁止，政令畅通，更好地担负起服务公民、服务社会、服务发展的责任。

加强廉政建设，深入开展反腐败斗争。认真开展保持共产党员先进性教育活动，创建学习型机关，不断提高公务员队伍素质和抓发展的能力，大力弘扬艰苦奋斗的优良作风，做到廉洁自律，勤政为民。积极开展对领导干部的经济责任审计，全面落实领导干部廉洁自律各项规定和廉政建设责任制，建立健全与社会主义市场n济体制相适应的教育、制度、监督并重的惩治和预防腐败体系，加大从源头上预防和治理腐败的力度。开展政府及其组成部门领导述廉评廉活动，自觉接受监督，堵塞管理漏洞，降低行政成本。深入开展廉政监察、执法监察，坚决惩处违法乱纪、以权谋私等腐败行为，努力消除“公共权力部门化、部门权力利益化、部门利益私人化”现象，以实际行动维护政府在人民群众中的形象。

各位代表，在新的一年里，改革发展的任务繁重而艰巨，我们的责任重大而光荣。机遇不可错失，形势催人奋进。让我们更加紧密地团结在以胡锦涛同志为总书记的党中央周围，认真贯彻邓小平理论和“三个代表”重要思想，在省委、省政府和中共邢台市委领导下，持之以恒地弘扬“树正气、讲团结、求发展”主旋律，持之以恒地营造“干事、创业、为民”的浓厚氛围，持之以恒地把各项工作“往深里想，往实处做”，把抢抓机遇的意识体现在精神状态上，体现在工作实际中，把实现更快更好发展的要求，体现在工作思路和工作措施上，体现在工作成效上，一项工作一项工作去做，一个难题一个难题去破解，解放思想，与时俱进，同心同德，扎实工作，努力开创邢台改革开放和现代化建设的新局面！
